# Salford Lung Study in chronic obstructive pulmonary disease (SLS COPD): follow-up interviews on patient-centred outcomes

**DOI:** 10.1038/s41533-017-0066-2

**Published:** 2017-12-15

**Authors:** Lynda Doward, Henrik Svedsater, Diane Whalley, Rebecca Crawford, David Leather, James Lay-Flurrie, Nick Bosanquet

**Affiliations:** 1 RTI Health Solutions, Manchester, UK; 2 Value Evidence & Outcomes, GSK, Brentford, Middlesex UK; 3 Global Respiratory Franchise, GSK, Uxbridge, Middlesex UK; 4 Clinical Statistics, GSK, Uxbridge, Middlesex UK; 5 Imperial College London, London, UK

## Abstract

This study investigated patient perceptions, experiences and management of COPD throughout the SLS COPD study. Follow-up interviews were conducted with 400 patients who completed SLS COPD; a mixed-methods approach was used to collect quantitative and qualitative information. Structured interviews using closed-ended questions were conducted with 360 patients, detailing aspects of background/lifestyle information and COPD. Extended interviews containing open-ended questions on perceptions of COPD and quality of life (QoL) in addition to the closed-ended questions were completed by 40 further patients. Participants also completed the Adherence Starts with Knowledge-12 (ASK-12) and the COPD and Asthma Sleep Impact Scale (CASIS) questionnaire. Quantitative data were analysed descriptively; qualitative data were analysed using qualitative description. The participants (*n* = 400) were reasonably representative of the SLS COPD population; mean age was 66.2 years. Breathlessness was the most commonly recalled symptom of/associated with COPD (88.5% of patients) and was the symptom that changed the most (improved, 26.8%/worsened, 20.9%) throughout the study. Participants’ daily functioning and activities were most affected by symptoms of/associated with COPD, followed by relationships and psychological issues. 66.5% of participants experienced exacerbations, 60.5% of whom reported self-management as their first treatment strategy (taking antibiotics, resting and/or corticosteroids). Qualitative analysis revealed COPD symptoms, breathlessness in particular, to have a significant impact on mobility and in turn QoL. In conclusion, breathlessness was cited in these interviews as the COPD symptom with the greatest impact on participants’ daily functioning, activities and self-care. The findings provided significant additional knowledge to the SLS COPD study findings.

## Chronic Lung Disease: Getting your breath back

Interviews with patients undergoing a clinical trial for chronic lung disease highlights the impact of breathlessness on quality of life. Henrik Svedsater at GlaxoSmithKline in the UK, and co-workers, wanted to dig deeper into patient perceptions of COPD during the Salford Lung Study in Chronic Obstructive Pulmonary Disease (SLS COPD) clinical trial, which trialed an alternative self-administered inhaler treatment. To this end, Svedsater’s team administered questionnaires and interviewed 400 patients involved in SLS COPD, to examine disease impacts on their quality of life. The patients cited breathlessness as having the greatest bearing on daily life, particularly on their ability to carry out physical tasks and continue a self-care regime. Investigations of disease impact should be incorporated into future clinical trials, particularly to assess how improving lung function affects patient perceptions and quality of life.

## Introduction

Chronic obstructive pulmonary disease (COPD) is one of the most common respiratory conditions in the UK, characterised by the chronic limitation of airflow due to, for example, obstructive bronchiolitis and emphysema.^1^ COPD commonly presents with an initial chronic cough and progresses to include sputum production and breathlessness (dyspnoea). Such symptoms present patients with a substantial physical burden, which may be further compounded by variable exacerbations.^1^ Despite this, people with COPD are often more concerned by the impact of their condition on their daily life rather than the symptoms themselves.^2^ However, perceptions of COPD impact are not frequently evaluated during clinical trials, and quality of life (QoL) assessments are not typically prioritised in clinical trial reporting.

Most clinical trials in COPD have been conducted using selected inclusion criteria, excluding co-morbidities for example, and so represent less than a quarter of people with COPD.^3^ Clear differences have been demonstrated in the characteristics of patients with COPD managed in primary care and those enroled in clinical trials, including age, gender, disease severity, QoL scores and exacerbation characteristics.^4^ Furthermore, it is well recognised that treatment strategies identified using such selected populations may fail to translate easily to populations managed in routine COPD care and thus complicate treatment decisions.^1^

The Salford Lung Study in COPD (SLS COPD) was a clinical trial conducted in a UK primary care population that was designed to be representative of patients with COPD in routine clinical practice.^5^ This open-label, randomised, controlled study investigated the effectiveness and safety of initiating treatment with the once-daily inhaled corticosteroid (ICS)/long-acting beta_2_-agonist (LABA) combination of fluticasone furoate (FF) 100 µg/vilanterol (VI) 25 µg compared with continuing usual care (UC) over a 12-month period. The rate of moderate or severe exacerbations over the 12-month study period, the primary outcome, was significantly lower in patients initiating treatment with FF/VI compared with continuing UC.^5^

We carried out follow-up interviews with a subset of patients completing SLS COPD, to record aspects of their experience not captured within the main trial and to further understand the impact of COPD on their lives. Here, we report the experiences of all SLS COPD follow-up interview participants together, while a subsequent manuscript will report analyses according to treatment group and key factors associated with higher rates of exacerbation, and further exploratory post-hoc analyses based on these data.

## Results

### Participant characteristics

Representativeness of the follow-up sample to the overall SLS COPD study sample was based on gender, age and exacerbation history (number of COPD exacerbations in the year prior to and during SLS COPD) (Table 1). Although interview participants were slightly younger than the overall study population, with a mean age of 65.2 years at the time of randomisation in SLS COPD vs. 66.8 years respectively (*p* = 0.002), there were no significant differences in gender or the number of exacerbations during SLS COPD. The characteristics of the 40 patients who participated in the extended interviews were also similar to those of the overall follow-up sample. More than half of participants in the follow-up sample were male (53.8%), in a relationship (56.3%) and retired (67%).

**Table 1:** Participant characteristics overall in SLS COPD and in the follow-up interviews (post-hoc)

	Total SLS COPD participants (*N* = 2600)	Follow-up interview participants (*N* = 400)	Extended interview participants (*N* = 40)
Demographic characteristics			
Mean age, years (SD)			
At start of SLS COPD	66.6 (9.8)	65.2 (9.3)	66.0 (8.4)
At entry into follow-up interview study	N/A	66.2 (9.3)	67.0 (8.4)
Male, *n* (%)	1322 (50.8)	215 (53.8)	21 (52.5)
Relationship status, *n* (%)			
Cohabiting^a^	N/A	225 (56.3)	24 (60.0)
Single or divorced/separated or widowed/surviving partner		173 (43.3)	16 (40.0)
Other		2 (0.5)	0
Employment status, *n* (%)			
Working full time	N/A	27 (6.8)	3 (7.5)
Working part time		13 (3.3)	0
Voluntary or charity work		5 (1.3)	2 (5.0)
Long-term sick leave		20 (5.0)	3 (7.5)
Retired		268 (67.0)	25 (62.5)
Unemployed or homemaker		49 (12.3)	3 (7.5)
Other		18 (4.5)	4 (10.0)
COPD exacerbations			
Number of exacerbations in year prior to SLS COPD, *n* (%)			
0	493 (19.0)	80 (20.0)	4 (10.0)
1	844 (32.5)	108 (27.0)	10 (25.0)
≥2	1263 (48.6)	212 (53.0)	26 (65.0)
Number of exacerbations during the SLS COPD, *n* (%)			
0	773 (29.7)	125 (31.3)	11 (27.5)
1	685 (26.3)	93 (23.3)	9 (22.5)
≥2	1142 (43.9)	182 (45.5)	20 (50.0)

*N/A* not available, *SD* standard deviation, *SLS COPD* Salford Lung Study in patients with chronic obstructive pulmonary disease

^a^Married/living as married/civil partnership

Only 10% (*n* = 40) of participants reported that they were working either full or part time, a further 0.8% (*n* = 3) were homemakers and 1.3% (*n* = 5) undertook regular voluntary or charity work; of these 41.7% (*n* = 20/48) reported that COPD directly interfered with their productivity or efficiency. Although the majority of participants (93.5% [*n* = 374]) reported no change in employment status over the study period, 14 participants (3.5% [*n* = 14]) reported that they had left work permanently or were on long-term sick leave. For six of those, COPD was the primary cause for this change in employment status.

Participants in the follow-up sample reported using a range of inhaled maintenance medications during the SLS COPD, which were selected from a pre-specified list. The most frequently used were ICS/LABA (fluticasone furoate/vilanterol [Relvar^®^], 52.8% [*n* = 211]; fluticasone propionate/salmeterol [Seretide^®^], 41.5% [*n* = 166]; budesonide/formoterol fumarate [Symbicort^®^], 7.3% [*n* = 29]) and tiotropium bromide (Spiriva^®^, 49.5% [*n* = 198]).

Health and lifestyle characteristics were also examined during the follow-up interviews (Table 2); 43.8% (*n* = 175) of participants had a long-term condition that limits physical activities or mobility in addition to COPD and 62.5% (*n* = 250) reported feeling down or depressed at least some of the time. More than half of the participants had given up smoking and more than a third were current smokers. Overall, 77.8% (*n* = 311) of participants reported taking some form of exercise, the most commonly cited being gentle aerobic exercise, flexibility and breathing exercises. Notably, a higher proportion of participants who undertook breathing exercises (84.4% [*n* = 119/141]) or high-intensity aerobic exercise (85.7% [*n* = 18/21]) reported that these helped their COPD when compared with those who undertook flexibility exercise (62.6% [*n* = 107/171]), resistance exercise (69.6% [*n* = 32/46]) or gentle aerobic exercise (78.4% [*n* = 174/222]).

**Table 2:** Participants’ health characteristics, including perceived control over COPD and lifestyle information reported in SLS COPD follow-up interviews

	Follow-up interview participants (*N* = 400)
Health characteristics	
Long-term illness or health problems in addition to COPD, *n* (%)^a^	
Condition that limits physical activities or mobility	175 (43.8)
Psychological or emotional condition	21 (5.3)
Cognitive condition	0
Other	116 (29.0)
None	148 (37.0)
Missing	4 (1.0)
How often feel nervous, anxious or panicky, *n* (%)	
None of the time	152 (38.0)
A little of the time	166 (41.5)
A lot of the time	61 (15.3)
All of the time	15 (3.8)
Missing	6 (1.5)
How often feel down or depressed, *n* (%)	
None of the time	143 (35.8)
A little of the time	153 (38.3)
A lot of the time	69 (17.3)
All of the time	28 (7.0)
Missing	7 (1.8)
Healthcare practitioner generally seen for COPD, *n* (%)^a^	
GP	360 (90.0)
Practice nurse	342 (85.5)
Hospital specialist	35 (8.8)
Respiratory nurse	60 (15.0)
Physiotherapist	5 (1.3)
Missing	1 (0.3)
Perceived control over COPD, *n* (%)	
Not at all	36 (9.0)
A little	120 (30.0)
Quite a lot	184 (46.0)
Very much	54 (13.5)
Not applicable	4 (1.0)
Missing	2 (0.5)
Change during SLS COPD, *n* (%)	
Improved a lot	77 (19.3)
Improved a little	53 (13.3)
No change	232 (58.0)
Got a little worse	14 (3.5)
Got quite a lot worse	21 (5.3)
Not applicable	2 (0.5)
Missing	1 (0.3)
Lifestyle information	
Smoking status, *n* (%)	
Currently smoking	148 (37.0)
[light; moderate; heavy]^b^	[50 (33.8); 65 (43.9); 29 (19.6)]
Given up smoking	215 (53.8)
Never smoked	25 (6.3)
Missing	12 (3.0)
Exercise undertaken, *n* (%)	
Any exercise	311 (77.8)
Breathing exercise^a^	141 (35.3)
Flexibility exercise^a^	171 (42.8)
Resistance exercise^a^	46 (11.5)
Gentle aerobic exercises^a^	222 (55.5)
High-intensity aerobic exercises^a^	21 (5.3)
Days per week of exercise, median (IQR), *n*	
Breathing exercise	7.0 (3.0–7.0), 140
Flexibility exercise	5.0 (2.0–7.0), 169
Resistance exercise	3.0 (2.0–7.0), 46
Gentle aerobic exercises	6.0 (3.0–7.0), 222
High-intensity aerobic exercises	3.0 (2.0–5.0), 21
Participants reporting that exercise helps COPD, *n*/*N* (%)^c^	
Breathing exercise	119/141 (84.4)
Flexibility exercise	107/171 (62.6)
Resistance exercise	32/46 (69.6)
Gentle aerobic exercises	174/222 (78.4)
High-intensity aerobic exercises	18/21 (85.7)

*COPD* chronic obstructive pulmonary disease, *GP* general practitioner, *IQR* interquartile range, *SLS COPD* Salford Lung Study in patients with COPD

^a^ Participants could select more than one response

^b^ Missing, *n* = 4 (2.7%)

^c^ For participants who reported undertaking this form of exercise

### Participant-centred outcomes

The nature and severity of participants’ COPD symptoms, and symptoms related to having COPD, are given in Table 3. Most participants (76.5% [*n* = 306]) recalled experiencing a COPD-related symptom in the 7 days prior to his/her interview. Of the pre-specified list of symptoms of COPD or associated with COPD, the most frequently recalled symptoms during the 12-month study period of SLS COPD were breathlessness (88.5% [*n* = 354]), phlegm (82.5% [*n* = 330]) and cough (79.3% [*n* = 317]). Breathlessness was also most frequently recalled, from the list of symptoms of or associated with COPD, as both the symptom that most improved (26.8% of patients reported an improvement [*n* = 95]) and most worsened (20.9% of patients reported a deterioration [*n* = 74]) during SLS COPD. Additional frequently recalled symptoms included tiredness or fatigue, dry throat and chest tightness. Many participants (57.5% [*n* = 230]) recalled that their COPD symptoms, and symptoms related to having COPD were worse when they felt anxious or upset. 62.3% of participants (*n* = 249) indicated that their COPD symptoms were worse at certain times of day, with morning and night-time being the most troublesome times (reported by 153 [38.3%] and 73 [18.3%] participants, respectively. Avoidance behaviour was commonly cited in an attempt to reduce symptom variability, with more than 80% of participants reporting a preference to avoid dusty, airless or overly warm environments.

**Table 3:** Recollection of symptom experience in SLS COPD

	Follow-up interview participants (*N* = 400)		
Overall symptom severity, *n* (%)			
Over the past 7 days	No symptoms	89 (22.3)	
	Mild	134 (33.5)	
	Moderate	128 (32.0)	
	Severe	39 (9.8)	
	Very severe	5 (1.3)	
	Missing	5 (1.3)	
Worse if upset/anxious	Yes, a lot	120 (30.0)	
	Yes, a little	110 (27.5)	
	No, not at all	138 (34.5)	
	Not applicable	28 (7.0)	
	Missing	4 (1.0)	
Worse at certain times of day^a^	Morning	153 (38.3)	
	Afternoon	19 (4.8)	
	Evening	44 (11.0)	
	During the night	73 (18.3)	
	None	148 (37.0)	
	Don’t know	2 (0.5)	
	Missing	1 (0.3)	
Symptoms of COPD or associated with COPD experienced during SLS COPD,^b^ *n* (%)	Participants	Change during SLS COPD	
		‘Most improved’^c^	‘Most worsened’^c^
Breathlessness	354 (88.5)	95 (26.8)	74 (20.9)
Cough	317 (79.3)	17 (5.4)	18 (5.7)
Phlegm	330 (82.5)	21 (6.4)	13 (3.9)
Chest tightness	218 (54.5)	13 (6.0)	7 (3.2)
Palpitations/panic attacks	124 (31.0)	4 (3.2)	2 (1.6)
Dry throat	260 (65.0)	4 (1.5)	8 (3.1)
Pain, aches/soreness	114 (28.5)	0	6 (5.3)
Headaches	97 (24.3)	2 (2.1)	4 (4.1)
Tiredness/fatigue	271 (67.8)	3 (1.1)	6 (2.2)
Sleep problems	193 (48.3)	1 (0.5)	8 (4.1)
Lack of appetite	96 (24.0)	0	3 (3.1)
Loss of bowel/bladder control	56 (14.0)	2 (3.6)	3 (5.4)
Other	20 (5.0)	0	2 (10.0)

*SLS COPD* Salford Lung Study in patients with chronic obstructive pulmonary disease

^a^ Patients could select more than one time of day

^b^ Based on a pre-defined list in the questionnaire

^c^ For patients reporting the corresponding symptom; patients could report more than one symptom as the most improved or worsened

Patients were asked to assess their current overall QoL on a scale of 1-to-10, where 1 = worst possible QoL and 10 = best possible QoL. The mean overall QoL score was 6.5 (standard deviation [SD] = 2.1 [*n* = 398]). Participants rated the effects of COPD on their functioning and activities as having the greatest impact on QoL (Table 4), with lifting or carrying, climbing stairs and other physical activities receiving the highest mean values (lifting or carrying, 2.6 [SD = 1.1]; climbing stairs, 2.9 [SD = 1.0]; physical activities, 2.7 [SD = 1.0]). Relationships and psychological well-being were also affected by COPD, to a lesser extent, and independence was the life area that was least affected by COPD for most participants.

**Table 4:** Effects of daily life impacts of COPD on five domains of participants’ quality of life (*N* = 400)

Functioning		Activities		Relationships		Well-being		Independence	
Reported impacts of COPD on aspects of daily life^a^; Mean score (SD), *n*									
Bending down	2.0 (1.0), *387*	Physical activities	2.7 (1.0), *371*	Relationship with partner	1.7 (0.9), *249*	Find coughing embarrassing	2.1 (1.1), *352*	Forced to plan activities	1.9 (1.1), *391*
Lifting/carrying	2.6 (1.1), *349*	Household jobs	2.2 (1.1), *376*	Socialising	1.9 (1.1), *337*	Get anxious/ worried	1.9 (1.0), *399*	Lost independence	1.7 (1.0), *399*
Walking outside	2.0 (1.0), *386*	Local shopping	1.9 (1.1), *302*	Holidays/days out	1.9 (1.1), *339*	Feel a burden on family	1.6 (1.0), *391*	Feel trapped in the house	1.6 (1.0), *399*
Climbing stairs	2.9 (1.0), *382*	Main shopping	2.2 (1.2), *266*	Helping/doing things with family	2.3 (1.0), *375*				
Talking	1.5 (0.8), *394*	Personal care	1.5 (0.9), *396*						
Effect of impact on QoL^b^; Mean score (SD), *n*									
	2.5 (0.9), *396*		2.5 (1.0), *397*		2.1 (1.0), *390*		2.2 (1.0), *392*		2.0 (1.0), *391*
Perceived change since the start of SLS COPD^c^; Mean score (SD), *n*									
	2.9 (1.0), *398*		3.0 (1.0), *394*		3.0 (0.8), *398*		3.0 (0.9), *393*		3.0 (0.9), *398*

*COPD* chronic obstructive pulmonary disease, *QoL* quality of life, *SD* standard deviation, *SLS COPD* Salford Lung Study in patients with COPD

^a^ Assessed as: 1, not at all; 2, a little; 3, quite a lot and 4, very much/unable to do

^b^ Assessed as: 1, not at all; 2, a little; 3, quite a lot and 4, very much

^c^ Assessed as: 1, improved a lot; 2 improved a little; 3, no change; 4, got a little worse; and 5, got a lot worse

The numbers in italic are *n* values

When probed further on the burden of COPD symptoms, and symptoms related to having COPD, half of the extended interview participants (*n* = 20) reported that breathlessness had the most impact upon their daily lives, most notably causing restrictions on mobility (Supplementary Table 1). Nine participants reported other COPD symptoms, such as phlegm and cough, as having the most impact. The inability to climb stairs was reported by a number of participants (*n* = 6) as a particular concern for their daily functioning. Notably, nearly half of the extended interview population (*n* = 19) highlighted these restrictions and limitations as the most important feature of COPD. Two participants commented on the embarrassment associated with other peoples’ perceptions of the condition. Six participants considered that COPD had no impact upon their lives.

Overall, 16.3% (*n* = 65/399) and 19% (*n* = 76/399) of participants perceived that their QoL had improved a lot or a little, respectively, during the SLS COPD study period. Additionally, 43.3% (*n* = 173/399) reported no change and just over a fifth of participants considered that their QoL had deteriorated by a large (6.5% [*n* = 26]) or a little (14.8% [*n* = 59]) amount.

### Perceived control of COPD, exacerbations and management

Participants’ perceptions of their control over COPD, exacerbations and self-management strategies are reported in Table 5. The vast majority felt they had ‘quite a lot’ (46% [*n* = 184]) or ‘very much’ (13.5% [*n* = 54]) control over their COPD, which remained relatively constant throughout the SLS COPD study period, with 58% (*n* = 232) of participants reporting no change in their level of control during the course of the study. However, approximately one-third of participants reported improvements of either ‘a lot’ (19.3% [*n* = 77]) or ‘a little’ (13.3% [*n* = 53]), and less than a tenth of participants reported deterioration of either ‘a lot’ (5.3% [*n* = 21]) or ‘a little’ (3.5% [*n* = 14]) since the start of SLS COPD (‘not applicable’ or missing answers applied to 0.75% of participants [*n* = 3]).

**Table 5:** Awareness and management of COPD exacerbations

Awareness of COPD exacerbations, *n* (%)	Follow-up interview participants (*N* = 400)		
Ever experienced exacerbation?			
Yes	266 (66.5)		
No	132 (33.0)		
Missing	2 (0.5)		
Severity of last exacerbation^a^			
Mild	21 (7.9)		
Moderate	74 (27.8)		
Severe	127 (47.7)		
Very severe	42 (15.8)		
Missing	2 (0.8)		
Aware when an exacerbation about to happen?^a^			
Yes	169 (63.5)		
No	97 (36.5)		
First course of action for most recent COPD exacerbation,^b^ *n* (%)	Managed at home *n* = 161 (60.5)	Sought medical help *n* = 99 (37.2)	
Activities			
Carry on your activities as you would normally do	17 (10.6)	12 (12.1)	
Pace yourself or do things differently	45 (28.0)	21 (21.2)	
Rest up completely	102 (63.4)	64 (64.6)	
None	0	2 (2.0)	
Missing	0	3 (3.0)	
Maintenance inhaler			
Carry on taking your maintenance inhaler as normal	138 (85.7)	74 (74.7)	
Increase the dose of your current inhaler	16 (9.9)	13 (13.1)	
Take your current inhaler more often during the day	9 (5.6)	12 (12.1)	
None	0	3 (3.0)	
Missing	0	3 (3.0)	
Additional medications			
Nebuliser	6 (3.7)	14 (14.1)	
Oral steroids	88 (54.7)	54 (54.5)	
Antibiotics	101 (62.7)	63 (63.6)	
None of the above	54 (33.5)	27 (27.3)	
Missing	1 (0.6)	3 (3.0)	
Seek medical help at any point? Yes / no	92 (57.1) / 69 (42.9)	99 (37.2) / 0	
Medical provider contacted or seen		Primary provider	Additional provider
GP	70 (76.1)	64 (64.6)	9 (9.1)
Practice nurse	6 (6.5)	7 (7.1)	0
Specialist doctor	1 (1.1)	0	0
Respiratory nurse	0.0 (0.0)	1 (1.0)	0
Urgent care service	0.0 (0.0)	5 (5.1)	0
Emergency service	16 (17.4)	23 (23.2)	4 (4.0)
Other	0	1 (1.0)	0
None	0	0	71 (71.7)
Missing	0	2 (2.0)	15 (15.2)

*COPD* chronic obstructive pulmonary disease, *GP* general practitioner

^a^ *n* = 266

^b^ Missing, *n* = 6 (2.3%)

Those patients who participated in the extended interviews were asked three open-ended questions relating to: their home supply of emergency medication; key exacerbation-onset warning signs; and exacerbation management decisions. The most frequently reported technique that participants used to self-manage their COPD symptoms, and symptoms related to their COPD, was ‘pacing yourself’ followed by planning activities, regular exercise and accepting aid for tasks. Self-management was also a popular strategy when tackling COPD exacerbations, a definition of which was provided in the interview schedule in order to ensure standardisation in patient understanding of the term. Approximately half of the participants (*n* = 199 [49.8%]) reported having a home supply of emergency medications or an emergency prescription. Of the 266 patients who had experienced an exacerbation, 60.5% (*n* = 161) of participants managed their last exacerbation at home by resting and/or self-medicating using antibiotics and/or oral steroids as a first course of action. The majority of the remaining participants sought medical attention either through their general practitioner (GP) (64.6% [*n* = 64/99]) or the emergency service (23.2% [*n* = 23/99]) to manage their last exacerbation. The medications that both groups used were similar; however, participants who initially sought medical attention were more likely to adapt their maintenance strategy than those whose first course of action was to manage at home. For the former group, 13.1% (*n* = 13/99) reported increasing the dose of their inhaled maintenance medication and 12.1% (*n* = 12/99) increased the frequency of maintenance inhaler use, while for the latter group 9.9% (*n* = 16/161) reported increasing the dose of their inhaled maintenance medication and 5.6% (*n* = 9/161) increased the frequency of maintenance inhaler use. Overall, 26/40 (65.0%) of extended interview participants had an awareness of an impending exacerbation, with 40% (*n* = 16/40) reporting that their first course of action was to manage their symptoms at home.

Extended interview participants reported three key strategies, which they employed to prevent worsening of symptoms: reducing or halting smoking, taking regular exercise and adhering to prescribed medication. Additional avoidance tactics were described by a number of participants, which broadly encompassed the evasion of particular environmental factors, such as smoke or dust, and refraining from undertaking heavily strenuous activities, such as running. Only two of the 40 participants interviewed reported not actively avoiding anything due to their COPD.

To assess barriers to adherence, participants completed the routine Adherence Starts with Knowledge-12 (ASK-12) scale. The median overall ASK-12 score was 1.7 (interquartile range [IQR] 1.4–2.0 [*n* = 399]), of a possible total of 5, where higher scores indicate greater barriers to adherence.^6^ The overall score comprised three domains: adherence behaviour, treatment beliefs and inconvenience or forgetfulness. Respectively the scores were: 1.2 out of 5 (IQR 1.0–1.4), indicating good adherence to prescribed therapy; 2.0 out of 5 (IQR 1.5–2.5), indicating that many participants had some confidence in their treatment;^5^ and 2.0 out of 5 (IQR 1.0–2.3), indicating that many participants did not find it difficult to take therapy as frequently as required.

### Recollection of relative symptom experience during SLS COPD

Symptoms that may be associated with having COPD (presented in the questionnaire as COPD symptoms, which may also be considered as adverse events [AEs]), that occurred during SLS COPD and were recalled by interview participants are detailed in Supplementary Table 2. The most commonly recalled symptoms in the standard interviews were tiredness/fatigue (67.8% [*n* = 271]), dry throat (65.0% [*n* = 260]), chest tightness (54.5% [*n* = 218]) and sleep problems (48.3% [*n* = 193]). This pattern was reflected in the extended interviews, where tiredness/fatigue, dry throat and chest tightness were the most frequently reported AEs. As tiredness/fatigue is quite a broad concept, it is difficult to determine whether it is a general reflection rather than representing a specific problem. No specific or serious AEs occurring after SLS COPD were reported.

## Discussion

### Main findings

The mixed-methods approach used for these follow-up interviews provided valuable insight into the perceptions and experiences of patients who completed the SLS COPD. Breathlessness was recalled as the dominant COPD symptom, while coughing, phlegm and chest tightness were also recalled as problematic symptoms. COPD had numerous impacts on participants’ QoL; breathlessness, in particular, appeared to have a significant impact on participants’ daily functioning and inhibited their ability to perform physical activities, such as walking up stairs, completing common household chores and engaging in self-care activities. The narrative provided by the qualitative data revealed that breathlessness also had the highest impact on participants’ QoL, regardless of whether participants recalled a change in this symptom during the course of SLS COPD.

### Interpretation of findings in relation to previously published work

This study provides a description of COPD experiences charted over time for a large number of patients, and in the context of an effectiveness trial set in clinical practice. The detailed evidence collected by our follow-up questionnaires provides further insight into the patient experience during SLS COPD by evaluating outcomes not traditionally examined in the context of a clinical trial. This study has highlighted the substantial impact of COPD on participants’ lives and the feelings of helplessness they experience, adding to knowledge gained from smaller-scale interviews that have been conducted with people who have COPD, such as the study of Svedsater et al.^2,7,8^ The impact of COPD on peoples’ lives is an often-overlooked outcome in clinical trial reporting, yet our interviews indicate that this is of crucial importance to those living with COPD. Our analyses also revealed that some people adapt to life with their condition better than others, taking preventative action such as avoiding environmental triggers and planning activities; supporting the importance of personalised treatment plans for COPD.^1^

Our finding that breathlessness had a significant impact upon participants’ daily lives and overall QoL was expected, as dyspnoea is known to be a major symptom of COPD, which directly impacts health-related QoL for people with this condition.^9,10^ Participants also reported diurnal variation in the severity of symptoms, which has been reported previously.^11,12^ An integrated COPD management approach (consisting of optimising patients’ COPD medication, encouraging physical activity, providing education and managing exacerbations) in primary care has been shown to improve QoL as a direct result of improving breathlessness.^13^ Participants in our study reported using similar techniques, such as gentle exercise and activity planning, to manage their condition.

Two-thirds of participants described having ‘quite a lot’/’very much’ control over their COPD during the 12-month SLS COPD study and a small group (*n* = 36) reported having ‘not at all’ control over their COPD, who could be considered as being at high risk of COPD complications. Similarly in another trial, more than 60% of patients reported having control over their COPD, despite also reporting that COPD negatively affected their life for at least 10 days per month.^14^ In the SLS COPD standard follow-up interviews, the first course of action for most participants during their last COPD exacerbation was to manage their symptoms at home. Reasons for the decision to seek medical help were discussed in the extended interview, and were determined by the failure of at-home strategies, the progression or severity of the COPD exacerbation symptoms and/or the lack of improvement over a specific time period. This ‘wait and see’ approach to seeking medical attention when managing exacerbations has been described previously in other COPD cohorts;^14^ Many participants reported completely resting or pacing themselves either to manage their COPD on a daily basis or in response to an exacerbation. These common coping mechanisms for COPD have also been observed within other study populations and termed ‘hiding’ and ‘battling’, respectively.^15^

Qualitative analyses revealed that although many participants were aware of strategies that might help prevent a worsening of their COPD, not all participants engaged in such strategies. Some participants considered that activities to improve lung function, such as exercise, exacerbated their COPD symptoms, and symptoms related to their COPD, and others denied the benefit associated with smoking cessation. This discord, between patients’ awareness of strategies to better manage their COPD and a refusal to carry out such behaviours, has also been identified in other qualitative studies; it has been suggested, particularly with regard to smoking, that patients may rationalise their current behaviour as it is difficult for them to acknowledge a responsibility for their condition.^16^

### Strengths and limitations of this study

The key strength of these follow-up interviews is the collection of participant-centred data following completion of the SLS COPD trial, which provides additional information that is not typically captured within the context of a clinical study, in addition to the SLS COPD study that aimed to reproduce the everyday clinical practice setting. As the participants had been well-characterised and charted throughout the duration of SLS COPD,^5^ our findings may be evaluated in context with the overall trial results and further explored in relation to clinical outcomes and standardised patient-related outcome assessments captured during the course of SLS COPD. Importantly, participants who completed the interviews were reasonably representative of the overall SLS COPD population. As SLS COPD was designed to recruit patients representative of the overall COPD population seen in routine clinical practice,^5^ the findings from the interviews could be applicable to a large proportion of patients with COPD. An additional strength of our approach is the use of qualitative description analysis, a valuable method of collecting information on individuals’ experiences in relation to a specific topic,^17^ which focusses on what the participants said, rather than extrapolating or making conceptual inferences.^18^ Follow-up questions were developed specifically for the study in order to provide information that goes beyond the standardised questionnaires that are typically included in clinical studies. A further strength of this study was the inclusion of a large number of patients in comparison with previous qualitative studies where typically fewer than 100 patients were interviewed.^15,19^

Limitations of the study include the exploratory and descriptive nature of the study design. It is also possible that a degree of self-selection occurred within the study population, as only those who had completed the SLS COPD study were eligible for inclusion in the interview, and not all invited individuals agreed to participate. In addition, the collection of open-ended data was limited to a subset of 40 participants from the overall sample. All participants in the study were recruited in one geographical area of the UK (Salford) and were English speaking, which may restrict the extrapolation of those particular findings to the wider COPD population. In the follow-up interviews, participants recalled their relative experiences of symptoms from a pre-specified list, which included direct symptoms of COPD (such as breathlessness) as well as COPD-associated symptoms (such as tiredness/fatigue). While these symptoms could be considered as recollections of AEs it should be noted that these recalled symptoms were not reconciled with the AEs recorded during the main SLS COPD trial as such data were not collected during the SLS COPD treatment period unless the events were serious and/or drug-related. It is also possible that some questions may have been influenced by recall bias (perception of changes in symptoms, function and well-being during the 12-month SLS COPD study period) or ranking bias (selective memory of the most debilitating symptom from the pre-specified list). However, the questions were carefully phrased to ask participants about change or progression over time, which are less likely to be affected by recall bias than questions that target specific events, activities or states.^20^ For example, in clinical practice physicians routinely ask patients about changes to their disease state over time to support care management and justify any changes in care treatment.^1^ Attribution bias may have also arisen in the interviews if participants attributed symptoms to their COPD that were caused by other underlying co-morbid factors, or vice versa. It is possible that the inclusion of symptoms commonly associated with COPD, rather than directly caused by COPD, in the pre-specified list of symptoms may have contributed to some degree of attribution bias in these interviews. Overall, we considered that the benefits of focussing the questions in these interviews on a specific dimension (aiding participant memory and reducing noise from potentially confounding conditions), to minimise recall bias, outweighed the risks of attribution bias. A similar approach to question design has been used previously for COPD patients, and no apparent recall bias was reported.^21^

### Implications for future research

Due to the qualitative nature of these results, our findings should be further verified beyond the present SLS COPD subpopulation. Assessment of COPD impact, particularly due to breathlessness, on the lives of people with COPD would be a valuable addition to future-planned clinical trials to identify how improvements in lung function relate to QoL. Further exploration of patient perceptions and self-management of exacerbations would also be an interesting avenue to examine in future studies with particular emphasis on self-management, avoidance strategies and overall impact within the wider COPD population.

## Conclusions

Breathlessness was the dominant symptom of COPD and the symptom that changed the most during SLS COPD. The greatest impact on QoL were the effects of COPD on participants’ functioning and activities. These results add valuable information to the main SLS COPD findings. A subsequent manuscript will compare the experiences of SLS COPD participants, stratified by the treatment group at randomisation, in addition to exploring predictors of exacerbations for these patients in post-hoc analyses.

## Materials and Methods

### Study population

In SLS COPD, patients aged 40 years or older, with a documented diagnosis of COPD, at least one COPD exacerbation in the last 3 years and history of regular maintenance inhaler therapy, were recruited between March 2012 and October 2014. Patients were randomly allocated to initiate treatment with either the FF/VI combination inhaler (100/25 μg; Relvar^®^/Breo^®^, GSK) or continue their normal COPD maintenance treatment (UC) as determined by their GP.

**Fig. 1:**
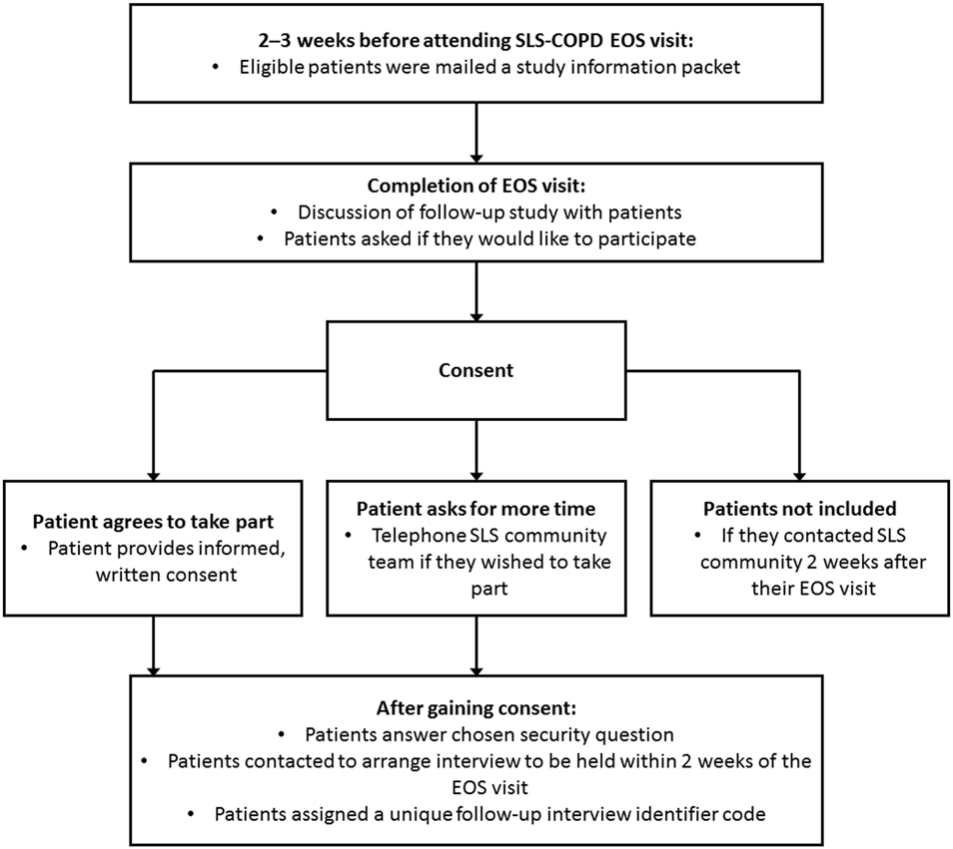
Patient recruitment process and consent. *EOS* end-of-study, *SLS COPD* Salford Lung Study in patients with chronic obstructive pulmonary disease

SLS COPD follow-up interviews were conducted with a subset of 400 patients completing the SLS COPD; interviews were conducted within 2 weeks of exit from the study. SLS COPD patients were recruited as illustrated in Fig. 1. Patients were deemed eligible for interview if they had attended the end-of-study visit, could participate in a qualitative interview with an English-speaking interviewer and could provide informed consent. Patients who provided written informed consent were invited to complete the follow-up interview within 2 weeks of their final study visit.

All 400 patients were asked a series of study-specific questions with multiple response options. A subset of 40 participants, selected using a simple random sampling process, were invited to participate in the extended interviews comprising a small number of open-ended questions, which were intended to explore selected issues in more detail. Initially, every 10th patient recruited into the follow-up study was invited to participate in these interviews. It was anticipated that such a random selection process would ensure that patients were sampled across different practices, demographic groups and treatment regimens. Following completion of 327 follow-up interviews (including 27 extended interviews), sampling for the extended interviews was increased to 1 in 7 patients to ensure that the quota of 40 extended interviews was met and 2–3 patients aged 80–90 years were purposively selected to address the shortfall in patients aged ≥80 years.

The SLS COPD follow-up interview study was approved by the Proportionate Review Sub-Committee of the Health Research Authority (formerly the National Research Ethics Service) East Midlands Research Ethics Committee in August 2013. Methods were performed in accordance with relevant regulations and guidelines.

### Study objectives

The primary objectives of this exploratory follow-up study were to determine the background and lifestyle characteristics of patients completing SLS COPD and to describe patient-centred outcomes beyond those captured by standardised instruments administered in SLS COPD. We focussed on aspects such as symptom experience, sleep, impact on daily life and overall QoL and collected participants’ experiences, perceptions and management of COPD, with a particular emphasis on disease awareness, self-management strategies and treatment-seeking behaviour. Additionally, we explored participants’ attitudes to medication and potential barriers to adherence.

### Questionnaires

#### SLS COPD follow-up interview schedules

A mixed-methods approach was used to collect both quantitative and qualitative information using questions developed specifically for the follow-up interview study. Quantitative data were collected via structured, closed-ended questions administered to all participants. Qualitative data were collected through semi-structured, open-ended questions on key topic areas administered to a subset of follow-up interview participants. The interview questions were derived from a brief targeted literature review and concept elicitation interviews conducted with 20 patients with COPD who were not members of the SLS COPD study cohort. This process identified key themes, which were then used to generate areas of questioning for the follow-up interview schedules. Two interview schedules were developed: firstly, a ‘standard’ interview schedule and, secondly, an ‘extended’ interview schedule.

The ‘standard’ interview questions used a variety of response options (yes/no or multiple response options). The closed-ended questions addressed: sociodemographic and lifestyle information; daily life impact of COPD across five domains (functioning, activity limitations, relationships, psychological impact and independence); COPD trigger factors (environmental and/or temporal); self-management of COPD and disease awareness; experience and management of COPD exacerbations; and QoL (overall and perceived change since study start). Interview participants were also asked to report on their recollection of their experience of COPD symptoms, and of symptoms associated with having COPD, during SLS COPD; this is described further below.

The ‘extended’ interview schedule included the same closed-ended questions from the standard interview plus an additional 14 semi-structured, open-ended questions. These were designed to explore in greater detail the areas addressed by the closed-ended questions, in addition to providing information on preferences for treatment outcomes.

Thus, the interview schedules were used to collect the same quantitative data for all follow-up study participants, while qualitative data were collected for a subset of participants selected at random from the overall COPD follow-up interview sample. An exacerbation was defined in the interview schedules as ‘an episode when your symptoms become much worse, and you need to change your treatment or you may need to seek medical help’. Both the standard and extended SLS COPD follow-up interview schedules underwent pilot testing with an additional sample of five COPD patients in order to assess their suitability and ease of use.

#### COPD and Asthma Sleep Impact Scale (CASIS)

All participants completed the CASIS questionnaire (scoring range 0–100, with higher scores reflecting a greater degree of impairment). The CASIS was developed as a measurement of sleep impairment associated with respiratory diseases such as COPD. It focuses on the experience of patients with either asthma or COPD (e.g., wake up at night with breathing problems), and excludes generic sleep deprivation (e.g., bad dreams, get up to use bathroom).^22^

#### ASK-12

All participants completed the ASK-12 questionnaire (scoring range 1–5, with higher scores indicating greater barriers to adherence). The ASK-12 total score identifies differences between groups of patients in terms of self-reported adherence indicators, including missed doses during the past week, the number of days medication was not taken as directed and treatment satisfaction.^6^

### Data collection

Questions were administered using the same format for both the standard and extended interview. Standard interviews were conducted by telephone or face to face if a telephone interview was declined. Extended interviews were conducted face to face. All interviews were performed by trained interviewers from the SLS follow-up interview team.

### Recollection of relative symptom experience during SLS COPD

Participants’ recollections of their experience of COPD symptoms, and of symptoms associated with having COPD, during SLS COPD were collected during all interviews. Specifically, patients were provided with a list of common COPD symptoms and associated symptoms; patients were then asked to recall whether they had experienced these symptoms with their COPD during the SLS COPD, and which of these symptoms had improved or worsened the most since the start of the SLS COPD. All selected symptoms that could be considered as AEs that were recalled by a participant during the follow-up interview, relating to a time during or after the main SLS COPD study period, were recorded. If the participant recalled a symptom that may be considered as an AE that occurred during the SLS COPD study period, the event was labelled as a ‘recollection of an SLS AE’. Events occurring after the SLS COPD study period were reported to the GSK Central Safety Department in accordance with ‘Spontaneous Adverse Events’ procedures.

### Statistical methods

Responses to the closed-ended questions were analysed descriptively using SAS 9.4 (SAS Institute INC.; Cary, North Carolina). The analysis of observed responses to individual questions was conducted by two independent programmers. The CASIS and ASK-12 questionnaire responses were analysed independently and scored according to standardised guidelines.^6,22^

A qualitative description approach^17^ was used to analyse the responses to the open-ended extended interview questions. Following primary and secondary coding of participant transcripts using the coding software ATLAS.ti (Version 7.1; Scientific Software Development, Berlin), a descriptive summary of participant-reported experiences and perceptions was produced for each theme analysed.

### Data availability

Access to the data sets supporting the conclusions of this manuscript may be obtained via https://www.clinicalstudydatarequest.com/.

## Electronic supplementary material

Supplementary Information
