# Supplementary material for: Salford Lung Study in chronic obstructive pulmonary disease (SLS COPD): follow-up interviews on patient-centred outcomes
Source: NPJ Prim Care Respir Med. 2017 Dec 15;27:66. doi: 10.1038/s41533-017-0066-2 (PMC5732268; doi:10.1038/s41533-017-0066-2)
Supplement: Supplementary file 1 — Supplementary Information [file 41533_2017_66_MOESM1_ESM.pdf]

SUPPLEMENTARY MATERIALS

Summary: Supplementary Table 1 contains a selection of illustrative quotes from the SLS COPD follow-up extended interview participants.

File type: Word document.

Supplementary Table 1. Selected participant quotes from the extended interviews

Topic	Extended interview participants' quotes (*N* = 40)
Symptoms	
Breathlessness, improved during SLS COPD	"I do occasionally get very breathless going up and down the stairs and if I was to walk from where I live into [Town] there is an incline and to come back home that incline can cause me to be breathless."
	"The breathlessness … I think really sort of getting upstairs or coming downstairs in a morning was difficult."
	"I will say the breathing [has the biggest impact] because it takes me all of my time. Well my husband usually makes the bed, you know the cover and that because I just get out of breath."
	"It's [breathlessness] improved a lot … Before I couldn't move without being really out of breath. It's improved my life a lot. I can get out more, I can do more."
	"I would say probably about 90% [improvement] it really has made such a difference I can actually go walking round the shops, I can go upstairs … unless it is like a thousand stairs or something. I go round the park with the girls when it's warm enough and it's quite different, usually I have to sit there while they play on the swings and things. I have been climbing on the frames and zip lines."
	"I can honestly say it's [breathlessness] better in the respect … The only way my breathing is affected mainly now is when I exert myself. No matter how small anything I try to do, even make myself a cup of tea and turn and move but normally, like I'm sat down now my

	breathing’s not affecting me. It’s no effect whatsoever.”<br>“I wouldn’t say it’s fantastic [improvement] but I can actually walk the length of the street which I couldn’t do before or I’d have to use the walking stick.”
Breathlessness, worsened during SLS COPD	“Well it’s just when I come to doing anything I’m out of breath … I try not to do it. I try not to walk to the shop. I get in the car. I live in a flat upstairs and I get to the top and I’m no breath. Where 12 months ago I’d have no problem.”<br>“Walking. If I decide to go for a walk anywhere I have to stop, you know, as 12 month ago, I did have to stop but nothing like I have to stop now.”<br>“I've noticed that, like going to bed up the stairs, now there's only 14 and a few years ago I used to be able cover them in... a couple of leaps and bounds, but now by the time I get to the top, I'm having to like gasp for air.”<br>“Yeah, it [breathlessness] varies. Some days I’m good and other days I’m not. I notice it more when I’m walking or I’m doing things in the house. Some days I can get that bit farther and on other days I only walk a couple of yards up street and I can feel it.”<br>“Changing clothing. I’m out of breath when I change my clothes.”<br>“It does restrict me for the length of time I can take to do a job, I think that is my biggest thing. I think oh well I will get this started I will do this today and I start doing it and I think I will do that and then I will go out and do such and such a thing, but I find that I am an hour or 2 hours behind myself because it has taken me longer to do it.”<br>“I mean there's certain things I can't do no more and I realise this and I miss it more with the grandchildren because I can't like, we've got one of two and I can't lift her up and carry her.”<br>“When I do get the breathlessness, it does frighten me.”<br>“I can only do a bit and then I have to stop and get my breath back.”

Cough, phlegm, chest tightness, improved during SLS COPD	"When I first got COPD me [sic] chest was always tight. I didn't think it was anything to do with COPD and stuff like that, but over the last 12–18 months it has eased off."
	"Well, I can walk down to the shop now … without getting breathless. And I can do housework and stuff now, compared to what I wasn't doing."
	"Well I have not had the cough which was annoying me because I was coughing that much before the study that was sort of like making my eyes hurt and that stopped."
	This individual regarded the reduction in cough as a great benefit of his participation in the study:
	"No coughing so that takes the strain off your chest and your lungs are rawness because you're constantly coughing and it hurts your eyes and it makes your head ache."
	"Well I could go up hills and I could go up and down the stairs and I didn't really notice it."
Other symptoms	"Oh yeah, it's [dry throat] a lot worse, 100% worse, because 12 months ago I didn't have it."
Symptoms that did not change during SLS COPD	"I think basically there has been no improvement there has not been any decrease in it but I think this is based on the standard of living I actually set myself."
Impact of COPD	
Life-impact	"When I get really breathless, that's scary. Your life flashes before your eyes, it's horrible."
	"It's every day of your life isn't it, it affects me every day, there's not a day goes by that it doesn't affect me."
	"It limits you, it limits your life. It's very limiting. You know I can see people worse than me but I can see people the same age as me that trot along quite brightly and I envy them, you know."

	“The worst thing really is not spending the time I should with my grandchildren.”
	“You wonder if you’re going to see tomorrow.”
	“Apart from the embarrassment of everybody knowing you’ve been smoking and done it to yourself I think that’s a really bad thought that, it really embarrasses me that.”
	“It doesn’t bother me. I just get on with life.”
Emotional impact	“It’s debilitating because you are already on a downer because of what you actually normally do in a sense and for it to be even more so it just makes you sad.”
	“Well the impact is that it depresses me, it is a bad impact.”
	“It can be very temperamental, it can hit me I can how can I put it, one day I can get up and I can feel that little bit better but the next day I feel down.”
Health interactions	“It’s a combination of both really that I can’t walk proper, so struggling to walk with my bad legs, so it’s putting a strain on my breathing.”
	“Well I always think my breathing, your heart failure, your breathing I think it’s all connected that.”
Diet	“I couldn’t breathe then because I was [overeating] and it wasn’t doing good for my weight and then it was like a vicious circle.”
	“Well I find that I get this unpleasant slimy like sensation at the back of my throat and if I have a fizzy drink that helps that.”
	“When I have a flare-up I don’t seem to eat as much.”
COPD management	
Prevention of	“I think the exercise does me good.”
COPD symptoms	“I try to do a little bit of walking each day.”
and symptoms	“I would like to do more exercises because I found that helps.”

related to COPD	“I know it’s [exercise] not going to do it, it’s just bluff... because you’re fooling yourself really … Well like I go in my wheelchair in here and I move the legs with my arms and I think to myself what the hell am I doing this for it’s not going to shift it.”
	“I mean I’ve tried exercise, it makes it worse.”
	“Well I think giving up smoking was the best thing that I ever did.”
	“I’ll tell you something it’s not made me feel any better which I thought quit smoking I feel exactly the same.”
	“It makes me worse if I stop smoking … I’ve tried and I don’t smoke as much.”
	“Sitting and doing nothing [can prevent worsening of COPD] but that doesn’t help in the long run.”
	“Well I think one of the main things is you watch your weight because that can affect you an awful lot more. It can you know have a much worse effect on your breathing and everything if you’re overweight and you’ve got COPD.”
	“Just take it easy, I think. Learn how to breathe properly when you know you’re going to get an attack.”
	“Not a lot I can do. If it gets worse it gets worse. That’s my opinion. I wouldn’t prevent it though.”
Avoidance tactics[1]	“I used to decorate and now I can’t put the paint because of the smell and things like that.”
Key exacerbation or onset warning signs	“I can tell when I start my phlegm gets thicker and thicker I can feel myself even when I have had a couple of puffs my puffer I am still panting a bit.”
	“An achy, a fluey-type, generally unwell, you know. Hot, miserable, really lethargic. I could sleep for England, if I start getting very tired and sleepy.”
Treatment decision making	“I start my own treatment when I’m convinced that’s what I need and I take them and providing it clears up I don’t go to the GP.”
	“I tend not to take any antibiotics until the colour of the phlegm changes.”
	“When I know it's not doing me any good, what I've done. If it's not improved with inhalers, make an appointment to see the doctor.”

	"Usually if I am coughing anything up and its changed colour, if it's green or then I will make an appointment."
	"When I can't do anything, When I can't even walk without getting out of breath."
	"When it starts to become really strenuous when everything becomes strenuous day-to-day life and things like that as well as the breathing side of things."
	"To get an appointment here is like gold."
Specific symptoms desired to improve with treatment	"My breathing obviously. No, I find this the biggest hindrance of the lot."
	"When it gets really tight. Yes I'd say the tightness."
	"Anything that would improve the quality of life that being whether it breathing and I am talking in a general term, because I believe that COPD we all know it's a killer, and if anything can be found to prolong the quality of life for any individual then I think that has got to be a priority."

SLS COPD Salford Lung Study in patients with chronic obstructive pulmonary disease

[1] For minimising or preventing COPD symptoms and symptoms related to COPD

Summary: Supplementary Table 2 reports the symptoms of COPD, or associated with COPD, that were recalled in the follow-up interviews and could be considered as adverse events during SLS COPD.

File type: Word document.

Supplementary Table 2. Recollection of relative symptom experience during SLS COPD

Recalled symptoms of COPD, or associated with COPD, that could be considered as AEs during SLS COPD,[1] *n* (%)	Follow-up interview participants (*N* = 400)		Extended interview participants (*N* = 40)	
	Experienced during SLS COPD	Reported as most worsened in SLS COPD[2]	Experienced during SLS COPD	Reported as most worsened in SLS COPD[2]
Chest tightness	218/400 (54.5)	7/218 (3.2)	24/40 (60.0)	0
Palpitations/panic attacks	124/400 (31.0)	2/124 (1.6)	16/40 (40.0)	0
Dry throat	260/400 (65.0)	8/260 (3.1)	28/40 (70.0)	1/28 (3.6)
Pain, aches or soreness	114/400 (28.5)	6/114 (5.3)	11/40 (27.5)	0
Headaches	97/400 (24.3)	4/97 (4.1)	8/40 (20.0)	1/8 (12.5)
Tiredness/fatigue	271/400 (67.8)	6/271 (2.2)	27/40 (67.5)	0
Sleep problems	193/400 (48.3)	8/193 (4.1)	15/40 (37.5)	1/15 (6.7)
Lack of appetite	96/400 (24.0)	3/96 (3.1)	8/40 (20.0)	0
Loss of bowel/bladder control	56/400 (14.0)	3/56 (5.4)	5/40 (12.5)	1/5 (20.0)
Other	20/400 (5.0)	2/20 (10.0)	2/40 (5.0)	0

AE adverse event, *SLS COPD* Salford Lung Study in patients with chronic obstructive pulmonary disease

[1] Based on a pre-defined list in the questionnaire

[2] For patients reporting the corresponding symptom; patients could report more than one symptom as the most worsened
